# “Extending the Technology Acceptance Model (TAM) to Predict University Students’ Intentions to Use Metaverse-Based Learning Platforms”

**DOI:** 10.1007/s10639-023-11816-3

**Published:** 2023-04-28

**Authors:** Ahmad Samed Al-Adwan, Na Li, Amer Al-Adwan, Ghazanfar Ali Abbasi, Nour Awni Albelbisi, Akhmad Habibi

**Affiliations:** 1 Department of Business Technology, Business School, Al-Ahliyya Amman University, Amman, Jordan; 2 Department of Educational Studies, Academy of Future Education, Xi’an Jiaotong-Liverpool University, Suzhou, China; 3 Translation and Interpreting Studies Department, Hamad Bin Khalifa University, Doha, Qatar; 4 Department of Management and Marketing, King Fahd University of Petroleum and Menerals, Dhahran, Saudi Arabia; 5 Department of Curriculum and Instructional Technology, Faculty of Education, University of Malaya, Kuala Lumpur, Malaysia; 6 Faculty of Education and Teacher Training, Universitas Jambi, Jambi City, Indonesia

**Keywords:** Metaverse, Higher education, TAM, Cyber risks, Enjoyment, Personal innovativeness, Self-efficacy

## Abstract

Metaverse, which combines a number of information technologies, is the Internet of the future. A media for immersive learning, metaverse could set future educational trends and lead to significant reform in education. Although the metaverse has the potential to improve the effectiveness of online learning experiences, metaverse-based educational implementations are still in their infancy. Additionally, what factors impact higher education students’ adoption of the educational metaverse remains unclear. Consequently, the aim of this study is to explore the main factors that affect higher education students’ behavioral intentions to adopt metaverse technology for education. This study has proposed an extended Technology Acceptance Model (TAM) to achieve this aim. The novelty of this study resides in its conceptual model, which incorporates both technological, personal, and inhibiting/enabling factors. The empirical data were collected via online questionnaires from 574 students in both private and public universities in Jordan. Based on the PLS-SEM analysis, the study identifies perceived usefulness, personal innovativeness in IT, and perceived enjoyment as key enablers of students’ behavioral intentions to adopt the metaverse. Additionally, perceived cyber risk is found as the main inhibitor of students’ metaverse adoption intentions. Surprisingly, the effect of perceived ease of use on metaverse adoption intentions is found to be insignificant. Furthermore, it is found that self-efficacy, personal innovativeness, and perceived cyber risk are the main determinants of perceived usefulness and perceived ease of use. While the findings of this study contribute to the extension of the TAM model, the practical value of these findings is significant since they will help educational authorities understand each factor’s role and enable them to plan their future strategies.

## Introduction

Over the last two decades, education changed as digital techology now used for teaching, learning, and assessment. (Dwivedi et al., 2022). Online learning environments like Blackboard and Moodle make asynchronous learning possible, while virtual meeting platforms like Zoom remove physical barriers to enable synchronous learning. These technologies has changed how students learn and instructors teach, creating new economic options for education providers and paving the way for AI-based adaptive learning systems (Kabudi et al., 2021). However, such learning technologies for online learning fall short of replicating in-person classroom experiences (Dwivedi et al., 2022). Creating virtual learning environments that meet course demands, learning goals, and provide high-level learning experiences is an unresolved issue. The concept of a three-dimensional virtual environment (3D) has emerged since the introduction of technologies such as Second Life in the market. Chandra and Leenders (2012) describe the virtual world as a digital multimedia 3D online environment that takes inspiration from reality and allows users to interact using avatars. According to Quintana and Fernandez (2015), virtual worlds have distinct features, including a three-dimensional format (making a more immersive experience than is the case with static images), active users’ role through the use of avatars, and a collaborative engagement with other users who also exist in the particular virtual environment through their avatars. The educational applications of virtual environments and related technologies have been the primary focus of previous research (Tang, 2021; Sebastien et al., 2018).

However, virtual environments for education cannot convey the cognitive and emotional experiences of engagement, gestures, co-presence, body language, and social contact. Consequently, they are unable to replicate the experience of tradetional learning (Dwivedi et al., 2022). Supporting this, it is found that virtual technologies such as virtual reality (VR), augmented reality (AR), extended reality (XR), and mixed reality (MR), do not, on their own, guarantee positive learning results (Marks & Thomas, 2022; Tegoan et al., 2021) due to issues related to the multisensory experiences of students in terms of teaching content quality and the appropriateness of realistic dynamic interactions. Therefore, the utilization of metaverse technology in education has the ability to enhance the online learning experience and make it simpler for educational service providers to establish virtual classrooms that imitate traditional classrooms (Teng et al., 2022). COVID-19 disrupted education and highlighted the importance of replicating in-person learning (Kim et al., 2022; Pappas & Giannakos, 2021). These acquired insights expedite educational reform and improve educational system readiness. According to Zhang et al. (2022), Immersive technologies like VR, AR, ER, and MR have increased interest in the metaverse, blurring the line between the virtual and physical worlds. Particularly, these technologies have contributed significantly in promoting metaverse in many educational applications (Tlili et al., 2022). Metaverse is widely seen that the Internet’s next generation, as it is expected to radically change how people interact and communicate with the world (Hwang and Chien, 2022). With the backing of the Internet of Things (IoT), artificial intelligence (AI), blockchain, and machine learning, metaverse technology can provide enhanced virtual and augmented reality experiences involving interactions between actual and virtual environments (Dwivedi et al., 2022). Metaverse has higher creativity, greater levels of customization, and lesser risk to promote student interaction, boost engagement and motivation and expand traditional learning activities by offering opportunities for experiences that might not otherwise be possible (Estudante & Dietrich, 2020).Thus, compared to previous technologies, the metaverse’s application in the area of education may better allow user-environment interaction, recreate emotional and cognitive processes, and more closely mimic the total face-to-face classroom experiences. Supporting this, it is found that metaverse-based learning platforms contribute significantly to increasing learners’ immersion and motivation (Akour et al., 2022). Metaverse enables students to attend virtual classes while still providing classroom-like elements. Students in a metaverse environment may use their avatars to interact with instructors and network with classmates.

From an educational standpoint, both industry and business need a well-educated workforce that can handle the innovative dilemmas of the metaverse settings. This, in turn, calls for new models of organizational and leadership management (Ahmad et al., 2021). Furthermore, Salloum et al. (2021) suggest that human behavior in the metaverse settings should be described and investigated in an educational environment in order to identify how it varies from behavior in the actual world. In a similar manner, education is among the most important and promising applications of the metaverse in the near future. In particular, HEIs could benefit from flexible platforms that allow instructors, students, and staff to interact without classroom limitations. In this way, the metaverse embraces actual learning environments (e.g., universities) by transforming them into a virtual environment in which instructors, students, and learning models may engage in collaborative and hybrid classrooms (Tarouco et al., 2013).

It is believed that the existence of the metaverse might well serve as a new educational setting (Prieto et al., 2022; Suzuki et al., 2020). The usage of the metaverse may be seen as an enhancement of education through the employment of metaverse-related technologies that integrate components of real and virtual educational environments (Zhang et al., 2022). In fact, through the metaverse, students and instructors will be able to engage in the virtual world while simulating the social and emotional spheres of the real world. The use of the metaverse for educational goals will enable students to interact easily with one another, with the instructors, and with the environment (Dwivedi et al., 2022). It will allow students to access the educational environment through the use of wearable devices without being restricted by time or place, and it will enable them to utilize digital identities (e.g., avatars) to engage in a real-time manner. This in turn will increase collaboration in learning activities, although still making independent learning possible (Teng et al., 2022). Despite being a virtual environment, it is nevertheless bound by physical constraints and has finite resources (Akour et al., 2022). The persistence side of metaverse-based learning platforms will allow the virtual environment to continue to exist and operate even after students log out of the metaverse platforms, and will allow them to retrieve any stored data when they rejoin.

Learner-centered education and problem-based learning may both benefit from metaverse-based learning platforms (Han, 2020). Problem-based learning is an excellent method for achieving learning goals (De Graaf & Kolmos, 2003). This method is particularly beneficial in the metaverse environment, where students, represented by avatars, must cope with a variety of issues. Educators provide learning cases that students, as avatars, must investigate and respond to in collaboration with other students, a process which may build their teamwork skills and increase their enthusiasm for learning (Farjami et al., 2011). Based on constructivist theory, the focus in the learning process should be on the students themselves, not the knowledge being taught (Bada & Olusegun, 2015). It is claimed that utilizing metaverse-based learning environments may facilitate learner-centered teaching by promoting both collaborative and independent learning (Suh & Ahn, 2022; Akour et al., 2022).

The educational implementation of the metaverse is still in its infancy due to the deficiency of implementation of AI-enabled adaptive learning systems and the IoT for immersive virtual-real space interactions (Dwivedi et al., 2022). In fact, the implementation of the metaverse in educational field entails several promises and involves several challenges for all stakeholders, including HEI policy makers, instructors, and students. It is crutial to evaluate how HEIs, which traditionally integrate both educational and social values, would adapt to meet the demands of students today. Adapting to the metaverse would ensure HEIs’ continued relevance in the digital era. If HEIs wish to increase the quality of their teaching services using metaverse technology, they will have to develop an adequate implementation. While top management and policymakers make decisions about how and when to deploy new technologies in higher education, the ultimate success of these initiatives significantly depends on students. Accordingly, for the metaverse to be widely and successfully adopted in higher education, it is obligatory to pinpoint the key factors that drive the adoption of the metaverse in higher education from the students’ standpoint. In Jordan, the use of educational technology has gained significant attention in recent years as a means of improving the quality and accessibility of education (Al-Adwan, 2020). However, there is a dearth of empirical research on the factors that influence the adoption of immersive technologies (e.g., AR, VR, metaverse) in higher education, particularly in developing countries such as Jordan (Faqih et al., 2021). In developing countries, there is a lack of widespread adoption of immersive technologies in organizations and among individuals due to a scarcity of adequate empirical studies on its use (Khan et al., 2019; Akçayır and Akçayır, 2017). The current study aims to identify and address any gaps in the existing research on the adoption of metaverse technology in higher education in Jordan. The study will also explore why Jordan has been slow to embrace metaverse technology, despite its long-standing interest in using digital technologies to support economic development. This analysis will focus on the most significant variables that impact the adoption of metaverse technology in Jordanian higher education from an educational perspective. Specifically, this study’s main objective is to determain the key factors that influence the behavioral intentions of higher education students to adopt metaverse-based learning platforms. Theoretical foundation and the suggested research model are presented in the next section. Next, the formulation of the research hypotheses is introduced. The findings will then be presented, followed by a discussion of the main findings. At last, the study’s implications and conclusions are reported.

## Theoretical foundation

The “Theory of Reasoned Action model” (TRA) by Fishbein and Ajzen (1975) is the foundation for the “Technology Acceptance Model” (TAM) (Davis et al., 1989). In fact, the TAM model was developed to identify the cognitive and psychological factors that influence users’ acceptance of new technologies. According to TAM, behavior intention (BI) is a determenant of technology adoption and use (Actual Behavior). Additionally, behavior intention is “jointly determined” by individuals’ attitudes toward technology use (A) and their perception of usefulness (PU). Individuals’ attitude is subsequently determined by their perceptions of usefulness (PU) and perceived ease of use (PEU). External variables determine both PU and PEU. As a result, TAM allows researchers to integrate extra potential factors that may drive the adoption of a specific technology through the external variable construct (Lee et al., 2019a, b).

According to Granić and Marangunić (2019), it has been widely established that TAM is among the most well-known models for predicting technology adoption and usage behavior. In particular, the TAM has witnessed an increased level of use by scholars for the purpose of predicting learners’ acceptance of learning technologies (Shen et al., 2022; Al-Adwan, 2020; Al-Emran et al., 2018). TAM, however, only offers broad insights into users’ willingness to adopt technology; hence, other factors that might impact a user’s technology adoption are necessary for context-based comprehension of the use of a particular technology (Zhang et al., 2022). Another criticism of TAM is that it focuses on technical aspects such as PU and PEU, and psychological aspects, such as BI, related to technology adoption, and overlooks users’ personal characteristics. Furthermore, while TAM considers extrinsic motivations (e.g., PU and PEO), it ignores intrinsic motivations (Taherdoost, 2018). Moreover, most of the TAM-related studies have ignored users’ negative (inhibiting) perceptions in favor of focusing only on their positive (enabling) ones (Dou et al., 2017; Cenfetelli, 2004). However, it has been claimed that it is critical to investigate the factors that lead to resistance behaviors among technology users (Kim & Kankanhalli, 2009). Researchers might possibly foster effective technology adoption and facilitate the adoption of novel technological innovation by examining the factors driving resistance (Roy et al., 2018). Thus, it seems pertinent and urgent to investigate the factors that facilitate and impede technology adoption. Accordingly, the original TAM constructs have been modified and extended by a large body of research by adding further variables, most of which may be divided into two main groups: (1) perceived variables, and (2) external variables (Al-Adwan & Berger, 2015).

**Fig. 1:**
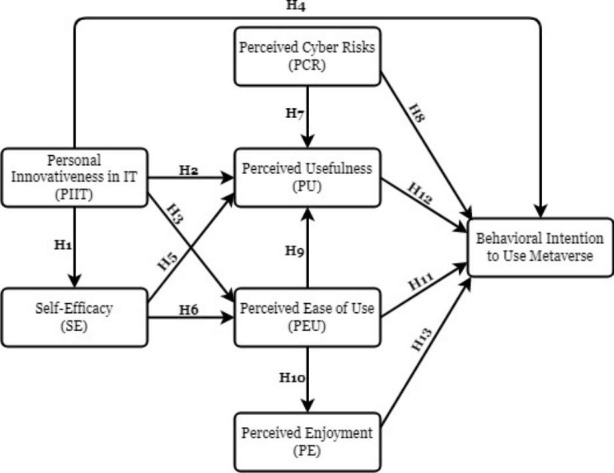
The research model

Our research used TAM because of its substantial empirical support in terms of its solid theoretical foundation, particularly for investigating the adoption of educational technologies in various contexts. Specifically, this study modifies TAM by incorporating perceived enjoyment (PE) (an intrinsic motivation) and perceived cyber risk (PCR) (an inhibitor) as direct predictors of the intention to use metaverse-based learning platforms, together with personal innovativeness in IT (PIIT) and self-efficacy (SE) as personal characteristics that act as key determinant of PU and PEU (see Fig. 1). PE reflects the level of joy and fun that can be obtained as a result of using a specific system or technology (Davis et al., 1992). It is claimed that PE significantly influences users’ intentions to adopt hedonic systems or technologies such as metaverse, as being ones which give users joy or pleasure (Humida et al., 2022; Van der Heijden, 2004). Supporting this, PE is recognized as a crucial variable in determining the use intention with regard to innovative technologies such as VR technology (Lee et al., 2019a, b) and AR technology (Cabero-Almenara et al., 2019). PIIT, as a personal trait, reflects an individual’s willingness to try out technologies (Agarwal and Prasad, 1998). Chen (2022) points out that individuals with high PIIT often seek out novel experiences based on cutting-edge IT products such as the metaverse. Several studies have confirmed the significant effect of PIIT on PU, PEU, and BI (Fagan et al., 2012; Yi et al., 2006). According to Ajzen (1991), SE designates the confidence that one possesses in one’s capability to engage in a specific behavior. The importance of SE as a key determinant of PU and PEU in the educational field is well-established in the literature of technology adoption (Chahal & Rani, 2022; Abdullah et al., 2016). Finally, PCR is incorporated into our research model in order to capture the effects of related risks (e.g., security risk, privacy risk) on metaverse adoption intentions. While the effect of PCR on metaverse adoption intentions has not been investigated, it is expected that mitigating PCR would positively influence metaverse adoption intentions (Sebastian, 2022).

## Literature review ans hypotheses development

### Personal innovativeness in IT (PIIT)

Personal innovativeness (PI) is the desire to try out new innovations (Fan et al., 2020). The notion of PI is extended to the field of IT (referred to as PIIT) to measure the degree to which an individual has an innate tendency to experiment with new information technologies (IT) (Agarwal & Prasad, 1998). According to Yi et al. (2006), individuals with high levels of innovativeness have a stronger tolerance for uncertainty and risk and are particularly open to trying out new ideas and changes. In the same vein, it is postulated that an individual’s personal innovativeness, which may be understood as a risk-taking tendency brought about by new technology, may have the most cognitive influence on how individuals make sense of information technology (Rogers, 2003). In addition, innovative individuals are identified as early adopters of innovation. The link between technology and the level of innovation receptivity affects how ready an individual is to adopt a technology (Agarwal & Prasad, 1998). Hence, individuals with higher IT innovativeness embrace change and have a great propensity to learn more about technological products. Additionally, they tend to have high technical skills that allow them to engage readily with technological products, which in turn boosts their favorable attitude towards products, causing them to focus their attention on the benefits of technological items and not be concerned about products performing as intended (Schweitzer & Van den Hende, 2016). In this study, PIIT describes the extent to which students have a strong propensity to try out metaverse in their learning. The extant literature confirms the significant effect of PIIT on PEU (or effort expectancy as an equivalent construct to PEU) in many contexts, such as personal digital assistants (PAD) (Yi et al., 2006), mobile learning (Joo et al., 2014), virtual learning (Raaij & Schepers, 2008), and healthcare technologies (Fan et al., 2020; Wu et al., 2011). Students who are more open to new technologies are inclined to embrace the use of metaverse-based learning and find it easy to use. Additionally, PIIT can lead to the development of new and innovative ways of using technology to enhance the learning experience, which can further improve the ease of use of metaverse-based learning. Moreover, PIIT is found to be a major enabler of PU (or performance expectancy as an equivalent construct to PU) in many contexts such as geotagging technology (Haque et al., 2020), online learning systems (Wang et al., 2021), and virtual reality simulation (Fagan et al., 2012). By being more receptive to new technologies, having a better understanding of how to use them effectively, and having a positive attitude towards learning through technology, students with high PIIT are inclined to find metaverse-based learning environments useful and beneficial for their educational needs. PIIT is also found as an vital enabler of the e-learning adoption intention (Chahal & Rani, 2022), and Chatbot adoption intentions (Trapero et al., 2020). Students who are more innovative in their use of technology are more open to new experiences, have greater computer self-efficacy, and perceive new technologies as useful and easy to use. These factors make it more likely that they will be interested in exploring and adopting new forms of technology-based learning, such as metaverse-based learning. Finally, it is claimed that high self-efficacy learners are also highly innovative because they are particularly open to new ideas, eager to use cutting-edge instructional media, and willing to try out new innovative digital technologies to satisfy their learning demands. Henece, by fostering an open and innovative mind-set towards new technologies, individuals can develop the skills and confidence they need to succeed in immersive and interactive virtual learning environments. Various researchers have confirmed the significant effect of PIIT on self-efficacy (Chahal & Rani, 2022; Bubou & Job, 2022). As a result, it is suggested that:

### Self-efficacy (SE)

According to Bandura (1986), SE represents individuals’ judgments in terms of “their capabilities to organize and execute a course of action required to attain designated types of performances” (p. 391). It is seen as an individuals’ belief in their own competence, and typically reflects the level of capability that an individual believes he/she possesses (Christensen and Knezek, 2015). According to Bubou and Job (2022), self-efficacy is intricately connected to self-esteem and self-worth, denoting a person’s level of confidence in a certain circumstance or event. It is also comparable to individuals’ self-confidence in their capability to deal with difficult situations. Given that self-efficacy is context-specific (Bandura, 1986), many researchers have investigated self-efficacy in different contexts, domains, and perspectives, such as computer self-efficacy (Nardi & Ranieri, 2019), internet self-efficacy (Joo et al., 2000), technology self-efficacy (Durak, 2018), and online learning self-efficacy (Hong et al., 2019). Technology self-efficacy, which is the main focus of this study, is defined as “personal belief in one’s ability to successfully use technology to increase learning outcomes” (Mikusa, 2015). Technology self-efficacy is vital factor for the successful use of an online learning system since it enables effective access to lecture materials, interaction with instructors and classmates, use of virtual instructional tools, discussion, and problem-solving (Durak, 2018). The current study employs self-efficacy to determine students’ judgment of their capability with regard to operating metaverse educational platforms to enhance their learning outcomes. Individuals with more confidence in their abilities to learn how to utilize information technology are inclined to view the technology as easy to use and beneficial, than those with less confidence (Venkatesh & Davis, 1996). Previous empirical findings have indicated that users with a high level of self-efficacy have robust perceptions of PU and PEU (Chahal & Rani, 2022; Thongsri et al., 2020; Fatima et al., 2017; Ibrahim et al., 2017; Abdullah & Ward, 2016). Hence, it is proposed that:

H5: “Self-efficacy (SE) has a positive influence on perceived ease of use (PU)”.

H6: “Self-efficacy (SE) has a positive influence on perceived usefulness (PEU)”.

### Perceived cyber risks (PCR)

Constant security concerns in online learning settings make it vital to examine how students perceive online learning related risks (Jiang et al., 2022). The perception of risk is often seen as the major barrier to user adoption of innovative technologies (Wang et al., 2018). In virtual worlds such as the metaverse, protecting user privacy and ensuring data security is an extremely important matter (Lee et al., 2021). Data in the metaverse serves as the primary form of governance, paving the way for collecting increasingly specific information from users (e.g., transactions, physical state, facial images) (Zhao et al., 2022). Despite the metaverse showing promise, the key challenges that hinder its sustained growth are issues related to security and privacy (Wang et al., 2022b). Managing massive streams of data, the prevalence of users’ profiling activities, and unfair outcomes of AI-based algorithms are just some of the potential security defects and privacy invasions that that have the potential to take place in the metaverse.

When it comes to user privacy in the metaverse, three major aspects stand out: personal information, behavior, and communications (Falchuk et al., 2018). Each of these aspects will supply significantly more data to metaverse platforms than is currently the case, resulting in increasing risks. In fact, this would enable the metaverse to expose more personal information with regard to users, not only on the platforms themselves, but also to other users. Di Pietro et al. (2021) point out that the metaverse’s current data gathering methods and accompanying analyses are regarded as amateurish. The metaverse platforms will be capable of tracking users’ private information such as physiological responses, body movements, as well as actual and virtual interactions with the surrounding environment. Importantly, the leakage of such personal information in the metaverse will lead to compromising a significant amount of data in terms of real world information about users’ physiological traits and habits. While such information is hard to acquire in the current Internet, it will be easy to access in the metaverse due to the closer connection between the virtual and real worlds in the future. This raises issues regarding user behavior privacy. In this aspect, the likelihood of real-world abuse and fraud in the metaverse’s online immersive interactions and experiences is high. Given that social engineering attacks currently make up the bulk of cyber-attacks experienced online (Salahdine & Kaabouch, 2019), it is anticipated that such attacks in the metaverse are expected to become considerably easier and more powerful, and hence more common (Di Pietro et al., 2021). With respect to the metaverse in education, computational problems are presented by the real-time collection and processing of interactive data between the virtual and physical worlds (Zhou, 2022). Furthermore, the information security of instructors, students, and others cannot be entirely assured. In addition, there is a greater chance of personal privacy leakage. It is also claimed that using anonymous logins may pose concerns and lead to violations. According to Zhang et al. (2022), owing to a greater degree of online anonymity in the metaverse, it will be easier for students with narrow social experiences to be subjected to criminal activities such as fraud, spying, and leaks. Once it occurs, learner privacy will be violated and might even negatively impact their daily lives. Additionally, both instructors’ and students’ creations and works run the danger of being plagiarized.

Previous research in various contexts indicates that perceived risk negatively influences not just behavior intentions with regard to technology, but also perceived usefulness, since it includes both negative consequences and uncertainty (Sarosa, 2022; Jiang et al., 2022; Trinh et al., 2020; Siyal et al., 2019; Wang et al., 2018). The less is users’ aversion to prospective expenditure and loss, the more they tend to adopt a specific technology. On the other hand, users who regard adopting a particular technology as a low-risk endeavor are likely to find it beneficial. However, the effect of PCR on BI and PU in the context of metaverse adoption in education has not yet been explored, which will be a key contribution of this study. Hence, it is argued that.

H7: “Perceived cyber risks (PCR) significantly and negatively affect perceived usefulness (PU)”.

H8: “Perceived cyber risks (PCR) significantly and negatively affect behavioral intention (BI) to use the metaverse”.

### Perceived ease of use (PEU)

PEU refers to the extent to which an individual perceives that using a given technology is easy and effortless (Davis et al., 1989). Previous studies on educational technology adoption indicated that users’ PEU substantially impacted their perception of usefulness (Fussell and Trouong, 2002; Mailizar et al., 2021). Moreover, such studies have shown that PEU is a major antecedent of educational technology adoption intentions Saleh et al., 2022a, b; Mensah et al., 2021). This study employs PEU to capture students’ perceptions of how easy and effortless it is to utilize the metaverse for learning purposes. Because educational metaverse platforms employ virtual reality and other forms of interactive technologies, information is presented in a manner that is as close to real life as possible, making it much simpler and easier to comprehend. In addition, educational metaverse platforms themselves are constructed in a manner that mimics the actual world. Users can acquire knowledge in a manner consistent with their past experiences, even while interacting in a virtual environment. Furthermore, it has been assumed that a strong relationship exists between PEU and perceived enjoyment (Davis and Bagozzi, 1992). Several previous researchers suggest that convenient and comfortable systems are more enjoyable (Wang et al., 2022a; Akdim et al., 2022; Tam et al., 2020). Consequently, it is anticipated that:

H9: “Perceived ease of use (PEU) significantly and positively affects perceived usefulness (PU)”.

H10: “Perceived ease of use (PEU) significantly and positively affects perceived enjoyment (PEN)”.

H11: “Perceived ease of use (PEU) significantly and positively affects behavioral intentions (BI) to use metaverse-based learning platforms”.

### Perceived usefulness (PU)

PU describes the extent to which individuals perceive that the usage of a specific technology enhances their performance (Davis et al., 1989). Earlier research has revealed that PU positively affects educational technology adoption intentions (Al-Rahmi et al., 2022; Akour et al., 2022; Al-Adwan et al., 2018, 2021; Martinho et al., 2018). PU in this study is described as the extent to which students perceive that the use of the metaverse will enhance their learning performance. The immersive and vivid learning experience provided by a metaverse-based leaning platform may enhance the quality of user engagement with virtual components. Thus, educational metaverse platforms help users to engage in learning activities successfully and effectively. Furthermore, users will have a more positive belief with regard to educational metaverse platforms since their learning effectiveness has been enhanced. Accordingly, it is proposed that:

H12: “Perceived usefulness (PU) significantly and positively affects behavioral intentions (BI) to use metaverse-based learning platforms”.

### Perceived enjoyment (PE)

Intrinsic motivation underpins the enjoyment concept (Abdullah & Ward, 2016), and is considered a hedonic motivation conceptualization (Venkatesh et al., 2012). PE represents the degree to which an individual has fun and pleasure as a result of utilizing a particular technology (Shen et al., 2022; Venkatesh, 2000, p.351) states that PE is the degree to which “the activity of using a specific system is perceived to be enjoyable in its own right, aside from any performance consequences resulting from system use”. In the educational field, PE establishes a connection between the playfulness and enjoyment of the student and the effectiveness and efficiency of the digital learning experience (Shen et al., 2022). Students are more likely to have a favorable opinion of a learning system and to have an increased intention to use it if they enjoy using it (Zhou et al., 2022; Esteban-Millat et al., 2018). Supporting this, previous research has confirmed the positive effect of PE on students’ behavioral intentions to use learning technologies Wang et al., 2022a; Zhou et al., 2022; Faqih et al., 2021; Cabero-Almenara et al., 2019). According to Barry et al. (2015), through the use of virtual three-dimensional environments and avatars, the metaverse piques students’ interest and motivates them to continue their learning. This is because the it makes learning more enjoyable, and because the environments in Second Life foster the instructors’ friendliness and students’ understanding. Accordingly, this study claims that intrinsically-driven students tend to use metaverse-based learning platforms for their learning. Thus, it is hypothisised that:

H13: “Perceived enjoyment (PE) significantly and positively affects behavioral intentions (BI) to use the metaverse”.

## Methodology

### Data collection and participants

This study investigates the intentions of higher education students to use metaverse technology in education. To accomplish the objective of this study, an extended TAM model was proposed and an empirical investigation was performed in the Jordanian context. While implementation of the metaverse in education still at an early stage, existing studies on virtual worlds in education allow us to reflect on the possible issues for investigating metaverse adoption in education (Dwivedi et al., 2022). Furthermore, the imaginary hypothetical settings for new and unknown innovative technologies such as metaverse technology, are notoriously hard to operate in (Schmitz et al., 2022; Yang et al., 2022). Consequently, the participants of this study were higher education students who have experience with interactive technologies (e.g., AR, VR, ER) and virtual worlds. These participants are in a better position to allow the researcher to achieve the objective of this study. However, the population for this study is recognized as indefinite; the sample frame of the targeted participants is absent. Hence, this study’s participants were recruited using the purposive sampling technique since it targeted students with experience in utilizing virtual worlds-related technologies. Purposive sampling entails recruiting participants if they meet particular screening conditions and based on their expertise or familiarity with the issue under investigation (Palinkas et al., 2015). As a result, 574 valid responses out of 967 participants were obtained from three public universities and two private universities in Jordan (see Table 1). As proposed by Hair et al. (2019), the adequacy of the sample size was determined based on the power analysis generated by G*Power software. This is a power analysis that takes into account the model structure, the expected effect sizes, and the anticipated level of significance (Memon et al., 2020). The calculation determines that the sample size is appropriate.

Data were collected from 11th June 2022 to 22nd September 2022 via the use of a web-based questionnaire. Specifically, the questionnaire was developed and hosted through the use of Microsoft Teams Survey Forms. With the assistance of academic colleagues in the five universities, the questionnaire link was shared on various online learning platforms (e.g., Moodle, Zoom, Microsoft Teams). Instructors were asked to send students frequent reminders with regard to completing the questionnaire to increase the response rate.

**Table 1:** Participants’ profile (N = 574)

Demographic		Frequency	%
Gender	Male	376	67%
	Female	188	33%
Age (Years)	< 20	145	26%
	20–30	298	53%
	> 30	121	21%
Education level	Under graduate	471	84%
	Post graduate	93	16%
University classification	Public	366	65%
	Private	198	35%
Specialty	Business and economics	169	30%
	Information technology	87	15%
	Engineering	54	10%
	Education	34	6%
	Tourism	62	11%
	Medical Sciences	51	9%
	Arts and Sciences	34	6%
	Law others	59	10%
	Other	14	2%

### Measures

Information about the respondent’s demographics is requested in the questionnaire’s first section, while the second part included 26 items in order to measure the constructs of the research model. Questionnaire items were adopted from previously published literature. Adjustments were made to these items to meet this study’s setting (Appendix A). A five-point scale from 1 “strongly agree” to 5 “strongly disagree” was used to assess each item. Both Arabic and English versions of the questionnaire were made accessible to respondents. We followed the back-translating procedure to ensure that the meanings of the questionnaire items remained consistent after being translated from English to Arabic (Brislin, 1970). The first draft of the questionnaire was pilot tested on 30 students to assess the internal consistency of each construct. The outcomes showed that each construct in the research model acquired an acceptable Cronbech’s Alpha (> 0.7) (Hair et al., 2019). Furthermore, a panel of experts consisting of five academics with a significant experience in educational technology and information systems was recruited to assess the content validity of the questionnaire (Artino et al., 2014). Based on the feedback of the panel, minor modifications were made to a few items.

### Data analysis

“The partial least squares structural equation modeling”- (PLS-SEM) technique was chosen for data analysis. In fact, PLS-SEM is a flexible approach that may be employed in a broad variety of settings, and whose sample size and distribution requirements are less conservative than those of other modeling techniques (Hair et al., 2019). We used the SmartPLS 4 software (Ringle et al., 2022) to analyze the data of this study. As instructed, we conducted the data analysis in two steps (Anderson & Gerbing, 1988). In step one, we evaluated the measurement model by assessing the internal consistency and convergent and discriminant validities. Since the data from the previous step were acceptable, we applied the structural model to verify our hypothesis in step two.

## Results

### Preliminary data analysis

The possibility of multi-collinearity and common method bias (CMB) were checked before the data analysis was started. For the purpose of assessing multi-collinearity, we used the “variance inflation factor - VIF”. Each VIF value must be < 3. (Hair et al., 2022). Consequently, there was no evidence of multi-collinearity since the VIFs varied from 1.549 to 2.868 (see Table 2). Harman’s single factor was then used to test for the existence of CMB. The finding showed that the loading of all measurement items in the dataset at once yielded a total variation of 44.678%, which is below the 50% threshold, indicating that CMB is absent (Podsakoff et al., 2003).

**Table 2:** Multi-collinearity assessment

Construct	BI	PEU	PU
PCR	2.186	-	1.986
PE	2.270	-	-
PEU	1.612	-	1.549
PIIT	2.868	1.617	2.623
PU	2.635	-	-
SE	-	1.616	1.730

### Measurement model

Before examining the proposed hypotheses, the reliability and validity of the measurement items (indicators) and scales (constructs) were tested (Hair et al., 2019). First, the loading of each indicator was assessed. A loading ≥ 0.708 indicates an acceptable item loading. Table 3 shows that the loading of each item is higher than the recommended value, suggesting that all items possess adequate item reliability. Second, two measures were used to evaluate the internal consistency: Cronbach’s Alpha (α) and composite reliability (CR). The minimum acceptable value of α and CR is recommended to be 0.7 and should not be ≥ 0.95. This condition is satisfied by all constructs (see Table 3), indicating that internal consistency is present in all constructs. Third, the convergent validity was determined by examining the “average variance extracted - AVE”. the minimum acceptable AVE value is 0.5. As can be seen in Table 3, the AVE value of each construct substantially exceeded 0.5, demonstrating that convergent validity exists in all constructs. In addition, the assessment of cross-loadings demonstrates that the items load substantially on their intended constructs, confirming the presence of convergent validity (Appendix B).

**Table 3:** Internal and convergent validity assessment

Construct	Item	Loading	“Cronbach’s Alpha (α)”	rho_A	“Composite reliability (CR)”	AVE
“Behavioral intention to use metaverse (BI)”	BI1	0.936	0.904	0.906	0.940	0.839
	BI2	0.910				
	BI3	0.902				
Perceived cyber risks (PCR)	PCR1	0.882	0.886	0.887	0.921	0.745
	PCR2	0.835				
	PCR3	0.875				
	PCR4	0.861				
Perceived enjoyment (PE)	“PE1”	0.852	0.878	0.0879	0.916	0.732
	“PE2”	0.871				
	“PE3”	0.846				
	“PE4”	0.853				
“Perceived ease of use (PEU)”	PEU1	0.899	0.900	0.901	0.930	0.770
	PEU2	0.879				
	PEU3	0.877				
	PEU4	0.853				
Personal innovativeness in IT (PIIT)	PIIT1	0.907	0.918	0.919	0.942	0.803
	PIIT2	0.894				
	PIIT3	0.890				
	PIIT4	0.892				
“Perceived usefulness” (PU)	PU1	0.899	0.921	0.924	0.944	0.809
	PU2	0.903				
	PU3	0.888				
	PU4	0.909				
self-efficacy (SE)	SE1	0.908	0.859	0.862	0.914	0.781
	SE2	0.862				
	SE3	0.880				

The last test at this stage was to examine discriminant validity. The √AVE of a construct should be greater that the construct’s correlation with any other construct. As demonstrated in Table 4, this condition is met, concluding that discriminant validity is present (Fornell & Larcker, 1981). Moreover, the result of the “Heterotrait–Monotrait Ratio-HTMT” test indicates that all HTMT values < 0.85 (Henseler et al., 2015), endorsing the findings in terms of Fornell and Larcker’s criterion.

**Table 4:** Discriminant validity

	BI	PCR	PE	PEU	PIIT	PU	SE
BI	**0.916**	0.733	0.764	0.564	0.788	0.811	0.682
PCR	-0.658	**0.863**	0.64	0.476	0.770	0.728	0.591
PE	0.681	-0.566	**0.855**	0.604	0.746	0.764	0.701
PEU	0.510	-0.426	0.538	**0.877**	0.626	0.580	0.555
PIIT	0.719	-0.696	0.670	0.569	**0.896**	0.764	0.693
PU	0.741	-0.658	0.688	0.529	0.703	**0.900**	0.674
SE	0.602	-0.517	0.608	0.488	0.617	0.600	**0.884**

Fornell–Larcker criterion (below the main diagonal) and Heterotrait–Monotrait Ratio (HTMT) (above the main diagonal).

Main diagonal: in bold, square root of the AVE.

### Structural model

After obtaining a satisfactory assessment of the measurement model, assessing the structural model is the next step. The significance of the path coefficients (β) was first assessed (Table 5). PU generated the strongest positive effect on BI (β = 0.345, p value < 0.001), followed by PIIT (β = 0.234, p value < 0.01), and PE (β = 0.217, p value < 0.01), demonstrating that these factors are key enablers of students’ metaverse adoption intentions. As expected, PIIT is found to be a key enabler of SE (β = 0.694, p value < 0.001), PEU (β = 0.465, p value < 0.001), and PU (β = 0.295, p value < 0.05), as it shows a significant positive effect on these factors. SE had a significant positive effect on PU (β = 0.212, p value < 0.01) and PEU (β = 0.232, p value < 0.001), indicating that SE is a key driver of PU and PEU. Unexpectedly, PEU exerted an insignificant effect on BI (β = 0.011, p value > 0.05), indicating that PEU plays an unimportant role in predicting students’ BI toward the adoption of the metaverse. However, PEU influenced both PU (β = 0.128, p value < 0.01) and PE (β = 0.604, p value < 0.001) positively and significantly, implying that PEU has an important role in boosting PU and PE. As proposed, PCR serves as a key obstacle of students’ BI with regard to metaverse adoption and with regard to PU, as it displayed significant negative effects on BI (β = − 0.158, p value < 0.05) and PU (β = − 0.315, p value < 0.01).

**Table 5:** Hypotheses testing

Hypothesis	Path	β	Mean	STDEV	T Statistics	Confidence interval	P Values	Assumption
H1	PIIT -> SE	0.694	0.694	0.035	19.967	[0.624, 0.761]	0.000	Yes
H2	PIIT -> PU	0.295	0.277	0.119	2.470	[0.015, 0.491]	0.014	Yes
H3	PIIT -> PEU	0.465	0.469	0.059	7.866	[0.356, 0.590]	0.000	Yes
H4	PIIT -> BI	0.234	0.226	0.089	2.630	[0.054, 0.407]	0.009	Yes
H5	SE -> PU	0.212	0.207	0.062	3.438	[0.093, 0.333]	0.001	Yes
H6	SE -> PEU	0.232	0.231	0.060	3.895	[0.116, 0.348]	0.000	Yes
H7	PCR -> PU	-0.315	-0.333	0.111	2.846	[-0.584, -0.155]	0.004	Yes
H8	PCR -> BI	-0.158	-0.171	0.080	2.032	[-0.367, -0.047]	0.030	Yes
H9	PEU -> PU	0.128	0.132	0.046	2.806	[0.044, 0.226]	0.005	Yes
H10	PEU -> PE	0.604	0.608	0.043	13.961	[0.524, 0.696]	0.000	Yes
H11	PEU -> BI	0.011	0.013	0.033	0.338	[-0.053, 0.076]	0.735	No
H12	PU -> BI	0.345	0.337	0.081	4.280	[0.174, 0.495]	0.000	Yes
H13	PE -> BI	0.217	0.218	0.074	2.926	[0.072, 0.366]	0.003	Yes

STDE: “Standard deviation”

With respect to predictive power (R^2^) (Table 6), the effects of PIIT, PEU, PU, PE, and PCR on BI yielded an R^2^ of 0.753, denoting that these factors explained a total of 75.3% of the variance in BI. Such an explanatory power is recognized substantial (Henseler et al., 2009). Furthermore, four factors (PIIT, PCR, PEU, and SE) contributed to explain 67.1% (R^2^ = 0.671) of the variance in PU, which is considered a substantial explanatory power. While the total variance explained in PEU is 41.9% (R^2^ = 0.419) in terms of SE and PIIT, PEU explains the total variance of 36.5% (R2 = 0.365) in PE. These explanatory powers are acknowledged as being moderate. Finally, a single factor (PIIT) explained a total variance of 48.1% (R^2^ = 0.481) in SE, suggesting a moderate explanatory power. The results of assessing the predictive relevance (*Q*^*2*^) are also displayed in Table 6. According to the results, all dependent variables had a predictive relevance value that is substantially greater than zero, implying that the research model has adequate predictive relevance (Hair et al., 2019).

**Table 6:** Assessment of predictive power and predictive relevance

Construct	R^2^	Assumption	*Q²*	Assumption
BI	0.753	Substantial	0.548	Large
PE	0.365	Moderate	0.209	Small
PEU	0.419	Moderate	0.270	Medium
PU	0.671	Moderate	0.476	Medium
SE	0.481	Moderate	0.295	Medium

In terms of assessing the effect size (*f*^*2*^), while the highest effect size on BI was generated by PU (0.143), PCR (0.120) produced the highest effect size on PU. Remarkably, the effect size of PIIT on SE was substantial (0.928) (see Table 7).

**Table 7:** Effect size assessment

Construct	BI	PE	PEU	PU	SE
PCR	0.036	-	-	0.120	-
PE	0.064	-	-	-	-
PEU	0	0.575		0.029	
PIIT	0.058	-	0.193	0.074	0.928
PU	0.143	-	-	-	-
SE	-	-	0.048	0.066	-

0.02, 0.15, and 0.35 depict small, medium, and large *f*^*2*^ effect sizes (Cohen, 1988)

### Indirect effect assessment

The significance of the indirect effects of the research model’s constructs is shown in Table 8. The results indicate that all indirect effects were significant with the exception of the indirect effect of PIIT and SE on BI through PEU. PIIT on PE produced the strongest indirect effect through PEU (β = 0.281, p value < 0.001), while SE generated the weakest on PU through PEU (β = 0.030, p value < 0.05). Interestingly, while PEU exerted an insignificant direct effect on BI (see Table 5), the indirect effect of PEU on BI through PE (β = 0.131, p value < 0.01) and PU (β = 0.044, p value < 0.05) were significant. This suggests that PEU can contribute to increasing students’ BI toward adopting the metaverse by improving PE and PU. Furthermore, the negative indirect effect of PCR on BI through PU (β = − 0.109, p value < 0.01) was significant, suggesting that PCR does not generate a negative direct effect on BI (see Table 5), but that it also adversely influences BI by decreasing PU.

**Table 8:** Indirect effects assessment

Path	β	Mean	STDEV	T Statistics	P Values
SE -> PU -> BI	0.073	0.070	0.027	2.672	0.008
PIIT -> PU -> BI	0.102	0.095	0.049	2.097	0.036
PEU -> PE -> BI	0.131	0.132	0.047	2.812	0.005
PIIT -> PEU -> BI	0.005	0.006	0.016	0.329	0.743*
PEU -> PU -> BI	0.044	0.044	0.018	2.426	0.015
SE -> PEU -> BI	0.003	0.003	0.008	0.319	0.750*
PCR -> PU -> BI	-0.109	-0.111	0.041	2.628	0.009
SE -> PEU -> PE	0.140	0.141	0.040	3.522	0.000
PIIT -> PEU -> PE	0.281	0.287	0.048	5.797	0.000
PIIT -> PEU -> PU	0.060	0.062	0.024	2.514	0.012
SE -> PEU -> PU	0.030	0.031	0.014	2.122	0.034
PIIT -> SE -> PU	0.147	0.144	0.045	3.288	0.001
PIIT -> SE -> PEU	0.161	0.160	0.041	3.892	0.000

*Insignificant effect

## Discussion

The findings demonstrate that PIIT significantly positively affects SE, PU, PEU, and BI, offering support for H1, H2, H3, and H4 respectively. Previous related research has found that PIIT is a key determining factor with regard to BI, SE, PU and PEU (Akour et al., 2022; Chahal & Rani, 2022; Bubou & Job, 2022; Wang et al., 2021; Fatima et al., 2017). This designates that highly innovative individuals are inclined to be more open to using new technologies, and realize that adopting them will not be particularly. Thus, high levels of PIIT encourage students’ BI to adopt metaverse-based learning platforms. In addition, students with a high level of PIIT tend to have positive SE beliefs, allowing them to successfully adopt new learning technologies, such as metaverse-based learning platforms. Students who possess a high degree of PIIT are inclined to approach challenges with enthusiasm, recognizing them as opportunities for personal development. Through the exploration of novel technologies and experimentation with their potential, these students can enhance their expertise and boost their confidence in effectively utilizing IT, such as metaverse. Moreover, high-innovative students encounter less complexity when using new educational technologies such as the metaverse due to their technical competency, which favorably influences their perceptions regarding using such technologies. In particular, such students are expected to find metaverse-based learning platforms easier to understand and operate than their peers. They are also often capable of benefitting from the potential benefits and related advantages of such platforms in their early stages of implementation.

The findings also reveal that SE positively affects both PU and PEU, indicating that H5 and H6 respectively are supported. These results are similar to those in previous research (Chahal & Rani, 2022; Thongsri et al., 2020; Fatima et al., 2017). High self-efficacy students are considerably more likely to accept new educational innovations and tend to have a highly favorable attitude regarding the usefulness of new technology such as metaverse-based learning platforms. When students have high levels of SE, they are inclined to perceive metaverse-based learning platforms as useful and easy to use. This is because they believe they have the skills and abilities necessary to succeed in the learning environment. Additionally, high levels of SE also lead to increased motivation and engagement, which can further enhance the perceived usefulness and ease of use of these platforms.

The results show that PCR exerts a significant negative effect on PU and BI, suggesting that H7 and H8 respectively are supported. This result recognizes PCR as a key inhibitor of metaverse adoption intentions, as it implies that the increased perceptions of cyber risk hinder students’ PU and BI in terms of the adoption of the metaverse. While the effect of PCR on metaverse adoption in education has not been investigated, it has been found to negatively affect PU and intentions in other technological contexts, including online learning (Sarosa, 2022; Jiang et al., 2022; Trinh et al., 2020; Siyal et al., 2019; Wang et al., 2018). Students’ personal information, behaviors, interactions, and habits are constantly collected and traced in the metaverse. Students perceive such acts as a source of privacy and security risk. When the perceptions of such risks are high, students will be inclined to avoid behaviors that endanger their personal information and invade their privacy, which obstructs the perception of usefulness and intentions to learn through the metaverse.

PEU significantly influences PU and PEN, indicating that H9 and H10 are supported. This implies that both PU and PEN will be inhibited if metaverse-based learning platforms are too complex to learn and operate and the user interface is unappealing. Such findings are similar to those of previous related studies in the area of educational technology (Wang et al., 2022a; Cabero-Almenara et al., 2019; Huang, 2019; Punnoose, 2012). If a metaverse-based learning platform is easy to use, students will be more interested in learning about its features and, as a result, will enjoy using it. When students encounter difficulties in using the metaverse platform, they may become frustrated and disengaged, which can lead to a negative experience. However, when students perceive the platform as easy to use, they are able to focus more on the engaging and enjoyable aspects of the learning experience.

Unexpectedly, the direct influence of PEU on BI is found to be insignificant, suggesting that H11 is not supported. This result is consistent with earlier research (e.g., Yang et al., 2022; Wang et al., 2022a), but it contradicts the findings of others (e.g., Akour et al., 2022; Faqih et al., 2021). This is probably because the respondants of this study are university students who belong to Generation Z (Z Gen) (born between 1995 and 2010 (Meet et al., 2022)). Individuals belonging to Gen Z are recognized as digital natives and as being tech-savvy, as they were born in the digital era and relied heavily on new technologies in their lives (Larionova et al., 2018). Thus, most of them have extensive knowledge and expertise in using modern technological solutions such as VR/AR equipment. As a result, they will tend to have few issues with regard to using and operating a metaverse-based learning platform easily.

Finally, as anticipated, PU and PEN exhibit significant effects on BI, indicating that H12 and H13 respectively are supported. While these results support those of earlier studies on the adoption of technology in education Wang et al., 2022a; Zhou et al., 2022; Faqih et al., 2021; Cabero-Almenara et al., 2019), they are inconsistent with others (e.g., Yang et al., 2022). The fact that metaverse-based learning platforms may have both an educational and entertainment impact is one explanation for these findings. Thus, students may hold both hedonic (e.g., perceived enjoyment) and utilitarian (e.g., perceived usefulness) values in high esteem. Students will be more eager to utilize a metaverse-based learning platform if they believe it will be exciting and beneficial to their education. When students find these platforms enjoyable and engaging, they are more likely to be motivated to learn, view technology in a positive light and be more willing to use it in their future academic endeavor. Additionally, if students perceive metaverse based learning platforms as useful, they are more inclined to adopt them. This is because they believe that using these platforms will enhance their learning outcomes and improve their overall academic performance.

## Implications

The contribution of this study is recognized to be twofold. First, the theoretical contribution is the development of a modified TAM model to explore the behavioral intention to adopt the metaverse in higher education. TAM has been widely criticized in terms of focusing on the technological and enabling aspects, and neglecting personal and inhibitor aspects. Thus, the study’s novelty stems from its conceptual model, which integrates technical, human, and inhibiting/enabling factors. This study validates the use of the TAM model and increases our understanding of metaverse adoption in higher education. The findings of this study add to the growing body of literature on metaverse technology. This technology has been the subject of a great deal of research, but its potential educational applications in higher education have received far less attention. As a result, our study enriches such knowledge and provides useful insight into the adoption of metaverse technology in higher education. The proposed model successfully explains 75.3% (R2 = 0.753) of the variation in metaverse adoption intentions (the outcome construct). This explanatory power is regarded as substantial. Furthermore, this research is thought to be one of the first to explore the vital factors that influence metaverse adoption intentions in higher education in Jordan. Thus, this study’s findings deliver useful insights that can be employed to support HEIs in Jordan in their efforts regarding a successful adoption of the metaverse. In addition to enhancing previous studies, this study may be used as a benchmark for similar investigations into the metaverse and offer implications for higher education in the future.

Second, the practical implications related to proposed crucial recommendations for education practitioners, developers, and designers of metaverse-based learning platforms in terms of the effective adoption of such platforms in higher education. The findings show that PU is a key facilitator of the metaverse. Thus, to increase perceived usefulness, it is important to explain how metaverse-based learning can enhance the learning experience compared to traditional methods. For example, highlight the potential for immersive and interactive learning, the ability to learn from a variety of sources, and the opportunity to collaborate with others. Metaverse developers may influence how students perceive their performance expectations by ensuring that metaverse-based learning platforms are significantly helpful when it comes to accomplishing the tasks necessary for learning more effectively and easily. In addition, the likelihood of students utilizing metaverse technology is likely to increase if it is shown to enhance learning and improve knowledge retention. In this regard, the utilisation of analytical tools (e.g., AI, big data, text mining) in metaverse-based learning settings may help measure, trace, gather, and analyse students’ learning data (e.g., behaviors, preferences, performances, and emotions). In light of this possibility, such data would not only assist instructors in comprehensively assessing students, but also might potentially provide students with tailored resources and services. Additionally, as students in the metaverse move from a static to a dynamic learning environment, and become the focal point of the teaching-learning process, it is necessary to develop new pedagogical and methodological paradigms compatible with the metaverse technology.

PEU is another crtical factor that facilitate the adoption of metaverse-based leraning. Hence, Thus, it is important to ensure that the metaverse-based learning platform is user-friendly and intuitive. If the platform is difficult to navigate or use, users may not perceive it as useful. Thus, it is essential to design the platform with user experience in mind, provide clear instructions and tutorials, and make sure that all the features are easily accessible. Furthermore, it is crutial to provide adequate training and support to ensure that users can effectively use the platform, it is essential to provide comprehensive training and support. This can include tutorials, videos, and online help systems to guide users through the platform’s features and functionalities. Moreover, optimize the platform for different devices is deemed essential. With the increasing use of mobile devices, it is crucial to ensure that metaverse-based learning platforms are optimized for use on different devices such as smartphones and tablets. This will make it easier for users to access the platform on-the-go.

PE is recognised as another facilitator of student intention to adopt the metaverse. Accordingly, students should be provided with enjoyable and engaging learning environments in which interaction is encouraged. Instructors should also focus more on how they deliver the curriculum in ways that promote student enjoyment. Instructors in particular should strive to students engaged in more enjoyable activities and emphasise their interactions with other students using metaverse-based learning platforms. While the effect of PEU on metaverse adoption intentions is found to be insignficant, PEU plays an important role in promoting metaverse adoption intentions by boosting PE and PU (key determinants of BI). Thus, to improve the perceptions of PEU, which in turn increases the perception of enjoyment and usefulness, it is crucial to build user-friendly interfaces for metaverse-based learning platforms and ensure their stability and dependability in operation. Utilizing the metaverse in education necessitates the availability of high-quality infrastructure tailored to common learning practices. The hardware and software designs and frameworks of the metaverse serve as the basis for educational activities. Multiple variables, including accessibility, safety, humanity, trust, educational capacity, and cognitive traits of students, should be included in the metaverse design for higher education administrators, instructors, and students. Additionally, special emphasis must be placed on education’s unique and extra design elements. For instance, smart wearable technologies (e.g., glasses, headsets) supported by high-speed networks like (e.g., 5G/6G) would allow students to access the metaverse world instantaneously, with no restrictions in terms of time or place. Importantly, it is vital to incorporate gamification elements into metaverse-based learning platforms. Gamification can be a powerful tool for increasing perceived enjoyment of use in metaverse-based learning. By incorporating game-like elements such as badges, leaderboards, and rewards, learners are more likely to feel a sense of accomplishment and enjoyment as they progress through the learning experience.

Furthermore, SE is realised as a key enabler for both PU and PEU. This suggests that curriculums should be revised to incorporate courses and training that will not only enhance students’ learning experience, but will also guarantee that future students will be able to effortlessly and seamlessly integrate new technology such as the metaverse, into their learning. In addition, instructors’ attitudes and knowledge regarding information technology may contribute to the formation of favorable students’ confidence, attitude, and enthusiasm toward using technology in their learning. Therefore, it would be advantageous to provide instructors with intensive training courses to help their students become more comfortable with, and proficient in, using technology, and metaverse technology in particular, in their learning activities. Instructors also need to create a supportive learning environment. Such an environment can help learners build their self-efficacy. Instructors can create a supportive learning environment by providing learners with the resources they need to succeed and by promoting a sense of community within the metaverse. In addition, it is fundamental to provide leaners with clear learning objectives. Learners with high self-efficacy tend to have a clear understanding of what they need to accomplish. Instructors can provide clear learning objectives for each module or task within the metaverse to help learners understand what they need to achieve.

PIIT has a significant role to play when it comes to metaverse adoption intentions as it has a direct and significant effect on SE, PU, PEU, and BI. Traditional learning tools and pedagogical strategies may have to be modified in order to satisfy the demands of students who constantly seek novel knowledge, learning methods, or ideas via interaction. In such a scenario, students will be able to adopt new technologies such as metaverse-based learning environments, and deal with a high degree of unpredictability in the real world. Additionally, given that learners with high PIIT tend to be more self-directed and have a strong desire for personalized learning, instructors should design metaverse-based learning experiences that allow learners to personalize their learning journey. This could include personalized learning paths, adaptive assessments, and individualized feedback. Instructors also are required to encourage risk-taking and failure. Personal innovators are willing to take risks and embrace failure as an opportunity to learn and grow. Instructors should create a safe and supportive environment that encourages learners to take risks and learn from their failures. This will help learners develop a growth mindset and become more resilient.

Importantly, PCR acts as a key inhibitor of PU and BI in terms of the metaverse. Therefore, it is imperative that strict regulations policies and rules be established and implemented in metaverse-based learning platforms. Consequently, there is a pressing need for regulators who perform the same role as the law enforcement authorities in the actual world, since it is anticipated that relevant laws and rules (such as real-name verification) would have to be put in place. As suggested by Zhang et al. (2022), traceability of creations, content, and works in the metaverse requires the employment of technologies such as blockchain and non-fungible tokens (NFT). If this does not happen, the metaverse may become a lawless environment, thus unsafe for students and instructors.

## Conclusion and limitations

An expanded TAM model was developed in this paper to investigate the main factors affecting higher education students’ behavioral intentions to adopt metaverse technology in their education. Based on 574 questionnaire responses collected from higher education students at five universities in Jordan, perceived usefulness, personal innovativeness in IT, and perceived enjoyment had substantial direct positive effects on students’ behavioral intentions. Furthermore, perceived cyber risk is recognized as an inhibitor, as it has significant negative direct and indirect (through perceived usefulness) effects on students’ behavioral intentions. The findings also indicate that the direct effect of perceived ease of use on behavioral intentions was insignificant, whereas its indirect effects through perceived enjoyment and perceived usefulness were significant. Importantly, the results highlight the critical role of personal innovativeness in IT as a facilitator of perceived usefulness, self-efficacy, and perceived ease of use. These findings can effectively help HEIs when it comes to designing and planning effective strategies for the successful adoption of metaverse technology for educational purposes. In particular, HEIs and metaverse developers should center their efforts on developing mechanisms to reduce the privacy and security risks associated with the use of metaverse technology.

It is important to note that our research has several limitations. The first constraint is the context of this empirical investigation. Jordan has various distinctive characteristics. Consequently, future studies should test the research model in different contexts. Scholars might compare the variations and commonalities in the contributing factors in diverse study settings. Another promising study avenue is to investigate instructors’ perspectives on the value and efficiency of metaverse technology. The empirical investigation of the research model from a temporal viewpoint might be another research route when digital technologies are implemented in education. Future research might go more into how metaverse technology is implemented and utilized. The research approach used in this study is another constraint. It is anticipated that experimental research methodologies will help assess their adoption’s degree and type of impact on learning, allowing for increased comprehension of the authentic influence of digital technology applications in education. Consequently, perception-based research is restricted in its comprehension of the effect. Future research initiatives using an experiment-based methodological approach might solve this problem. Finally, a future avenue to pursue is that of exploring potential enhancements to the proposed study model by incorporating moderating variables (e.g., disciplines, age, gender) in order to increase its effectiveness.

## Data Availability

The datasets used and/or analyzed during the current study are available from the corresponding author on reasonable request.

## Appendix A. Questionnaire

**Table:**

Construct	Item	Source
Perceived Usefulness	PU1: “Metaverse educational platforms enable me to accomplish tasks more quickly”.	Yang et al. (2022); Teng et al. (2022); Faqih et al. (2021)
	PU2: “Using a metaverse educational platform enhance my learning effectiveness “	
	PU3: “Metaverse educational platforms enhance the quality of my learning”.	
	PU4: “Metaverse educational platforms are useful in tertiary tourism education”.	
Perceived Eease of Use	PEU1: “Learning to use/operate a metaverse educational platform would be easy to me”.	Shen et al. (2022); Teng et al. (2022); Noble et al. (2022)
	PEU2: “It is easy for me to become skillful at using a metaverse educational platform”.	
	PEU3: “I find that the use of a metaverse educational platform is not complicated/does not require a lot of mental effort”.	
	PEU4: “My interaction with metaverse educational platform is clear and understandable”	
Self-efficacy	SE1: " I am confident that I can perform effectively on many different tasks”	Luo & Du 2022
	SE2: " When facing difficult tasks, I am certain that I will accomplish them.“	
	SE3: " I believe I can succeed at most any endeavor to which I set my mind “	
Perceived Enjoyment	PE1: “Using a metaverse educational platform in learning is fun”.	Shen et al. (2022); Faqih et al. (2021); Lee et al. 2019a, b
	PE2: “Using a metaverse educational platform in learning is enjoyable”.	
	PE3: “Using a metaverse educational platform in learning is very entertaining”.	
	PE4: “Metaverse devices will make my leisure time more fun”.	
Perceived Cyber Risks	PCR1: “The personal information that enters into a metaverse educational platform will be handled securely”.	Saleh et al., 2022a, b;
	PCR2: “Metaverse educational platforms have enough safeguards to make me feel comfortable using them to access educational services”.	
	PCR3: “I am concerned that Metaverse educational platforms distribute the gathered data without my knowledge to third-parties”.	
	PCR4: “I perceive Metaverse educational platforms as more privacy-invasive compared to non- Metaverse educational platforms”.	
Personal innovativeness in IT	PIIT1: “If I heard about a new information technology, I would look for ways to experiment with it”.	Fan et al. (2020); Yi et al. (2006)
	PIIT2: “Among my peers, I am usually the first to try out new information technologies”.	
	PIIT3: “In general, I am hesitant to try out new information technologies.“ (Reverse question)	
	PIIT4: “I like to experiment with new information technologies”.	
Metaverse behavioral intention	BI1: “Intend to use a metaverse educational platform for my studies in the future”.	Shen et al. (2022); Teng et al. (2022)
	BI2: “I predict I would use a metaverse educational platform for my learning experiences”.	
	BI3: “I plan to use a metaverse educational platform frequently”.	

## Appendix B. Cross-loading

**Table:**

	BI	PCR	PE	PEU	PIIT	PU	SE
BI1	**0.936**	-0.608	0.612	0.468	0.673	0.656	0.542
BI2	**0.910**	-0.576	0.643	0.463	0.632	0.667	0.517
BI3	**0.902**	-0.622	0.617	0.469	0.671	0.712	0.594
PCR1	-0.593	**0.882**	-0.491	-0.364	-0.651	-0.586	-0.438
PCR2	-0.575	**0.835**	-0.521	-0.387	-0.572	-0.571	-0.485
PCR3	-0.583	**0.875**	-0.496	-0.379	-0.614	-0.575	-0.459
PCR4	-0.514	**0.861**	-0.442	-0.34	-0.560	-0.538	-0.399
PE1	0.582	-0.514	**0.852**	0.509	0.616	0.601	0.515
PE2	0.592	-0.48	**0.871**	0.464	0.559	0.609	0.542
PE3	0.548	-0.467	**0.846**	0.437	0.554	0.568	0.552
PE4	0.607	-0.474	**0.853**	0.426	0.562	0.574	0.472
PEU1	0.48	-0.403	0.481	**0.899**	0.535	0.467	0.426
PEU2	0.439	-0.359	0.457	**0.879**	0.490	0.450	0.443
PEU3	0.455	-0.391	0.487	**0.877**	0.488	0.498	0.429
PEU4	0.411	-0.339	0.461	**0.853**	0.483	0.438	0.414
PIIT1	0.617	-0.625	0.605	0.514	**0.907**	0.632	0.565
PIIT2	0.666	-0.633	0.593	0.516	**0.894**	0.674	0.585
PIIT3	0.650	-0.594	0.624	0.513	**0.890**	0.594	0.541
PIIT4	0.641	-0.641	0.579	0.496	**0.892**	0.617	0.518
PU1	0.671	-0.585	0.603	0.48	0.616	**0.899**	0.545
PU2	0.678	-0.605	0.624	0.492	0.651	**0.903**	0.528
PU3	0.664	-0.593	0.641	0.471	0.628	**0.888**	0.550
PU4	0.653	-0.585	0.606	0.458	0.636	**0.909**	0.536
SE1	0.556	-0.493	0.521	0.443	0.585	0.553	**0.908**
SE2	0.497	-0.437	0.524	0.412	0.523	0.523	**0.862**
SE3	0.541	-0.438	0.568	0.439	0.527	0.513	**0.880**

## Notes

The original online version of this article was revised: The author–s name “Nour Awni Albelbisi” was misspelled as “Nour Awni Albelbis” in the original publication of this article.
